# Association between the dietary index for gut microbiota and gallstone disease: A cross-sectional analysis considering the Dietary Inflammatory Index

**DOI:** 10.1097/MD.0000000000049813

**Published:** 2026-07-31

**Authors:** Wenwen Ni, Changqing Deng, Yijin Chen

**Affiliations:** a Department of General Surgery, Deqing People’s Hospital (Deqing Campus, Sir Run Run Shaw Hospital, School of Medicine, Zhejiang University), Deqing, Zhejiang, China; b Department of Gastroenterology, Meizhou People’s Hospital, Meizhou Academy of Medical Sciences, Meizhou, China.

**Keywords:** diet, dietary index, gallstone disease, gut microbiota, NHANES

## Abstract

The dietary index for gut microbiota (DI-GM) reflects dietary patterns that may support or adversely affect gut microbial health. This study examined the association between DI-GM and prevalence of gallstone disease (GSD), while additionally exploring the potential mediating association of the Dietary Inflammatory Index (DII), using data from the National Health and Nutrition Examination Survey 2017–2020. This cross-sectional study analyzed 5669 adults from National Health and Nutrition Examination Survey 2017–2020. GSD status was defined by self-reported physician diagnosis. DI-GM scores were calculated from 14 dietary components categorized as beneficial or detrimental to gut microbiota. DII scores were derived from nutrient intake data. Associations were evaluated using multivariable logistic regression. Higher DI-GM scores were associated with a lower likelihood of GSD (adjusted odds ratio [OR] = 0.92, 95% confidence interval [CI]: 0.87–0.97, *P* = .004). Individuals with DI-GM ≥ 6 had significantly reduced GSD risk (adjusted OR = 0.71, 95% CI: 0.55–0.91, *P* = .007). The beneficial dietary component was inversely associated with GSD (adjusted OR = 0.93, 95% CI: 0.86–0.99, *P* = .047). No significant interactions were observed across subgroups. Mediation analysis showed an inverse association between DI-GM and DII, with a small but significant indirect association (approximately 28%). Higher DI-GM scores are associated with a lower prevalence of GSD. These findings suggest a potential role of dietary inflammatory in this association; however, given the simultaneous measurement of exposure, mediator, and outcome, the results should be interpreted with caution.

## 1. Introduction

Gallstone disease (GSD) is a prevalent gastrointestinal disorder characterized by the formation of solid deposits within the gallbladder and may lead to complications such as cholecystitis, pancreatitis, and biliary obstruction.^[1]^ In the United States, GSD are present in approximately 10% to 20% of adults, leading to over 700,000 cholecystectomies annually and incurring healthcare costs exceeding $6 billion.^[2]^ Risk factors for GSD include advancing age, female gender, obesity, rapid weight loss, pregnancy, and certain genetic predispositions.^[3]^ Dietary habits also play a significant role, with diets high in cholesterol and low in fiber increasing the risk of gallstone formation.^[4]^

The gut microbiota plays a pivotal role in bile acid metabolism and cholesterol homeostasis.^[5]^ Furthermore, the gut microbiota influences gallbladder motility through immunomodulatory mechanisms.^[6]^ Dietary patterns significantly influence the composition and function of the gut microbiota. Several studies have investigated the relationship between dietary quality indicators and GSD prevalence. For instance, a study utilizing data from the 2017–2020 National Health and Nutrition Examination Survey (NHANES) examined associations between dietary quality indicators and GSD, highlighting that patients with GSD exhibited poor dietary habits.^[7]^ Another study explored the associations between the Dietary Inflammatory Index (DII) and Composite Dietary Antioxidant Index with GSD prevalence, suggesting that diets with higher inflammatory potential and lower antioxidant capacity may increase GSD risk.^[8]^ Additionally, research has linked higher DII scores to increased GSD prevalence, emphasizing the role of diet-induced inflammation in GSD pathogenesis.^[9]^ As our knowledge, there is no study investigating the association between the dietary index for gut microbiota (DI-GM) and GSD risk in US adults.

Recent mediation analyses indicate that the DII may partly account for associations between dietary exposures and disease risk. For example, studies in lifestyle research have reported that diet–disease relationships, including those related to mental health and cognitive outcomes, may be partially explained by dietary inflammatory potential.^[10]^ In the context of GSD, these findings suggest that DII may contribute to the observed association between diet and disease risk.^[11]^ However, the relationship between dietary patterns reflecting gut microbiota-related diet quality, such as the DI-GM, and GSD has not been evaluated in a nationally representative population. The DI-GM is a recently developed index that captures overall diet quality in relation to gut microbiome health.^[12]^ By examining the association between DI-GM scores and GSD prevalence, this study aims to provide epidemiologic evidence on the role of diet quality in GSD among U.S. adults. The findings may help inform dietary guidance and public health strategies aimed at reducing the burden of GSD.

## 2. Methods

### 2.1. Study design and population

This cross-sectional study utilized data from the NHANES cycles 2017–2020 (https://wwwn.cdc.gov/nchs/nhanes/continuousnhanes/default.aspx?Cycle=2017-2020). NHANES is a program designed to assess the health and nutritional status of adults and children in the United States through interviews and physical examinations. The survey employs a complex, multistage probability sampling design to ensure a representative sample of the civilian, noninstitutionalized U.S. population. For the present analysis, we used the NHANES 2017–March 2020 pre-pandemic dataset, and appropriate sample weights were applied according to NHANES analytic guidelines. In practice, this entailed dividing each 2-year interview or Mobile Examination Center exam weight by 2 (e.g., WTMEC4YR = 0.5 × WTMEC2YR) to obtain a 4-year weight. For dietary intake, we used the 2-day recall weight WTDR2DPP (2017–2020 cycle) and similarly divided it by 2 to form the combined 4-year dietary weight (WTDR4YR). The NHANES protocols were reviewed and approved by the National Center for Health Statistics Research Ethics Review Board. All NHANES participants provided written informed consent prior to data collection, acknowledging voluntary participation and understanding of the study purpose, procedures, potential risks, and benefits. This study is a secondary analysis of publicly available, de-identified NHANES data; therefore, no additional institutional review board approval was required.

### 2.2. Assessment of GSD

GSD status was determined using self-reported data from the NHANES medical conditions questionnaire. Participants were asked if they had ever been told by a doctor or other health professional that they had gallstones. Those who answered affirmatively were classified as having GSD. In this study, we excluded participants who were under 20 years old, had incomplete GSD questionnaires, or lacked data on dietary quality indicators, resulting in a final sample of 5669 individuals.

### 2.3. Dietary assessment and DI-GM and DII calculation

Dietary intake data were collected using two 24-hour dietary recalls. The first recall was conducted in person at the Mobile Examination Center, and the second was conducted via telephone 3 to 10 days later. These recalls captured detailed information on all foods and beverages consumed by participants during the preceding 24 hours.

The DI-GM includes 14 dietary components: 10 considered beneficial and 4 deemed unfavorable to gut microbiota health (Table S1, Supplemental Digital Content 1). The beneficial components are avocado, broccoli, chickpeas, coffee, cranberries, fermented dairy products, dietary fiber, green tea, soybeans, and whole grains. The unfavorable components are red meat, processed meat, refined grains, and high-fat diets (where fat constitutes 40% or more of total energy intake).^[12]^ Each component was scored 1 or 0, based on its consumption relative to sex-specific median intake values calculated separately for males and females using weighted NHANES 24-hour dietary recall data, accounting for the complex survey design. These medians were used as data-driven cutoff points for DI-GM scoring. The individual scores for all components are summed to obtain the overall DI-GM score, which ranges from 0 to 14 (Table S2, Supplemental Digital Content 2). Higher DI-GM scores indicate dietary patterns more conducive to fostering a healthy and diverse gut microbiome.^[12]^ Participants were stratified into 4 groups according to the quartiles of their total DI-GM scores: 0 to 3, 4, 5, and ≥ 6.

Dietary intake data were obtained from two 24‑hour recalls collected in the NHANES 2017–2020, and individual intakes of nutrients, food items and bioactive compounds relevant to the DII algorithm were estimated. The DII algorithm is grounded in literature linking food components to 6 inflammatory biomarkers (CRP, IL‑1β, IL‑4, IL‑6, IL‑10, and tumor necrosis factor alpha) and comprises up to 45 dietary parameters such as total energy, macronutrients (protein, carbohydrate, fat), micronutrients (vitamins A, C, D, E, B6, B12, folate, magnesium, zinc, selenium, and iron), fatty acids (saturated, monounsaturated, and polyunsaturated), fiber, cholesterol, flavonoids, and specific food groups.^[13]^

For each parameter, individual intake was standardized by subtracting a global referent mean and dividing by the referent standard deviation to produce a *Z*‑score, which was then converted into a centered percentile score (multiplying by 2, then subtracting 1) so that the distribution ranged approximately from –1 to + 1. After this transformation, each centered percentile score was weighted by its respective inflammatory effect score (derived from literature linking that component with inflammatory biomarkers) and all weighted scores were summed to produce the total DII for each participant. Higher (more positive) DII values reflect a more pro‑inflammatory dietary pattern, and lower (more negative) values reflect a more anti‑inflammatory pattern.

### 2.4. Covariates

In this study, several covariates were included to control for potential confounding factors in the analysis. These covariates were selected based on their relevance to GSD prevalence and dietary patterns. Age was treated as a continuous variable, while gender was categorized as male or female. Ethnicity was classified into 4 groups: Hispanic, Non-Hispanic White, Non-Hispanic Black, and Other. Marital status was divided into 2 categories: married/living with a partner or other. Poverty income ratio was calculated based on household income and family size, with categories defined as < 1.30 (low income), 1.30–3.50 (middle income), and ≥ 3.50 (high income). Smoking status was recorded based on participant self-reports of current smoking behavior. Hypertension and diabetes status were determined through self-reported diagnoses by a healthcare provider or measured during health assessments conducted by NHANES. Height was measured with a stadiometer and weight with a calibrated scale, then body mass index (BMI) was calculated as weight (kg) divided by height squared (m^2^).

### 2.5. Statistical analysis

All statistical analyses accounted for the complex survey design of NHANES by incorporating sample weights, stratification (SDMVSTRA), and clustering (SDMVPSU), as recommended in the NHANES Analytic Guidelines. Descriptive statistics were used to summarize participant characteristics, stratified by GSD status. Logistic regression models were employed to examine the association between DI-GM scores and GSD. Unadjusted models were first conducted, followed by models adjusted for potential confounders, including age, sex, ethnicity, BMI, smoking status, hypertension, and diabetes. Descriptive statistics and regression models were computed using survey-weighted methods. For example, survey-weighted *t*-tests and Rao-Scott chi-square tests were used for group comparisons, and survey-weighted logistic regression (R’s survey package) was applied to estimate associations, reporting odds ratios (ORs) with 95% confidence intervals (CIs). This approach uses Taylor series linearization for variance estimation, properly reflecting the multistage design. To assess potential effect modification, stratified analyses were conducted by age group, sex, ethnicity, smoking status, and BMI categories. Interaction terms were included in the models to test for statistical significance of interactions. The total, direct, and indirect effects were estimated, and the proportion of the effect mediated by DII was calculated. Mediation analyses were performed using the “mediation” package in R software. Analyses were performed using R (version 4.x) with the survey package. A two-sided *P*-value of <.05 was considered statistically significant. To evaluate the robustness of the findings, several sensitivity analyses were conducted. First, subgroup analyses were performed across major participant characteristics. Second, missing covariate data were handled using multiple imputation by chained equations, generating 5 imputed datasets under the missing-at-random assumption, with pooled estimates calculated using Rubin rules. Third, analyses were repeated without applying NHANES sampling weights to assess the influence of survey weighting. Fourth, models were additionally adjusted for total energy intake, alcohol consumption, marital status and physical activity in sensitivity analyses.

## 3. Results

### 3.1. Characteristics of the study participants

A total of 5669 participants were included in the study, with 602 (10.6%) having GSD. Participants with GSD were significantly older (58.39 ± 15.01 years) compared to those without GSD (47.86 ± 17.87 years, *P* < .001). GSD prevalence was higher among females (70.8%) than males (29.2%; *P* < .001). There were significant differences in ethnicity distribution, with a higher proportion of Non-Hispanic Whites (44.5%) among those with GSD compared to other ethnic groups (*P* < .001). Participants with GSD had a higher prevalence of hypertension (56.5% vs 35.0%, *P* < .001) and diabetes (28.7% vs 13.1%, *P* < .001) compared to those without GSD. Additionally, the mean BMI was significantly higher in the GSD group (32.56 ± 7.34 kg/m^2^) compared to the non-GSD group (27.56 ± 5.28 kg/m^2^; *P* < .001). The DI-GM score was lower in participants with GSD (4.68 ± 1.83) compared to those without (4.90 ± 1.65, *P* = .002). The beneficial to gut microbiota component was significantly lower among those with GSD (2.13 ± 1.54 vs 2.27 ± 1.3, *P* = .013), while the unbeneficial component showed no significant difference (*P* = .07; Table 1).

**Table 1:** Characteristics of the study participants, NHANES (2017–2020).

Characteristic	Overall (N = 5669)	Non-GSD (N = 5067)	GSD (N = 602)	*P*-value
Age (yrs)	48.98 (17.88)	47.86 (17.87)	58.39 (15.01)	<.001
Gender				<.001
Male	2792 (49.3%)	2616 (51.6%)	176 (29.2%)	
Female	2877 (50.7%)	2451 (48.4%)	426 (70.8%)	
PIR (%)				.068
<1.30	1569 (27.7%)	1407 (27.8%)	162 (26.9%)	
1.30–3.50	2176 (38.4%)	1920 (37.9 %)	256 (42.5%)	
≥3.50	1924 (33.9%)	1740 (34.3%)	184 (30.6%)	
Ethnicity (%)				<.001
Hispanic	1183 (20.9%)	1037 (20.5%)	146 (24.3%)	
Non-Hispanic White	2127 (37.5%)	1859 (36.7 %)	268 (44.5%)	
Non-Hispanic Black	1418 (25%)	1295 (25.6%)	123 (20.4%)	
Other	941 (16.6%)	876 (17.3%)	65 (10.8%)	
Marital (%)				.501
Married/living with partner	3355 (59.2%)	2999 (59.2%)	356 (59.1%)	
Other	2311 (40.8%)	2065 (40.8%)	246 (40.9%)	
Smoking status (%)				.547
Smoker	1386 (25.2%)	1247 (25.5%)	139 (23.3%)	
Nonsmoker	4109 (74.8%)	3652 (74.5%)	457 (25.5%)	
Hypertension (%)				<.001
Yes	2109 (37.2%)	1769 (35%)	340 (56.5%)	
No	3553 (62.8%)	3291 (65%)	262 (43.5%)	
Diabetes (%)				<.001
Yes	838 (14.8%)	665 (13.1 %)	173 (28.7%)	
No	4831 (85.2%)	4402 (86.9%)	429 (71.3%)	
Body mass index (kg/m^2^), mean ± SD	28.63 (6.15)	27.56 (5.28)	32.56 (7.34)	<.001
DI-GM, mean ± SD	4.88 (1.67)	4.9 (1.65)	4.68 (1.83)	.002
Beneficial to gut microbiota, mean ± SD	2.26 (1.33)	2.27 (1.3)	2.13 (1.54)	.013
Unbeneficial to gut microbiota, mean ± SD	2.62 (1.01)	2.62 (1.02)	2.54 (1)	.07
Dietary Inflammatory Index (DII)	1.29 (1.83)	1.23 (1.79)	1.76 (1.97)	<.001
Physical activity (%)				.026
Active	4732 (83.5%)	4287 (84.6%)	445 (73.9%)	
Inactive	937 (16.5%)	780 (15.4%)	157 (26.1%)	
C-reactive protein (mg/L)	3.12 (5.41)	2.87 (5.16)	4.68 (6.47)	<.001

All continuous variables are presented as means (standard deviations) and categorical variables presented as numbers (%). *P*-values were calculated based on independent sample *t*-test or Chi-square test.

DI-GM = dietary index for gut microbiota, GSD = gallstone disease, NHANES = National Health and Nutrition Examination Survey, PIR = poverty income ratio.

### 3.2. Association between DI-GM and GSD

Table 2 presents the association between DI-GM and GSD. In the unadjusted model, a higher DI-GM score was inversely associated with GSD (OR = 0.92, 95% CI: 0.87–0.97, *P* = .002). This association remained significant after adjusting for covariates: OR = 0.92, 95% CI: 0.87–0.97, *P* = .004). When stratified by DI-GM categories, participants with a DI-GM score ≥ 6 had a significantly lower likelihood of GSD (crude: OR = 0.68, 95% CI: 0.54–0.86, *P* = .002; adjusted: OR = 0.71, 95% CI: 0.55–0.91, *P* = .007), with a significant trend across categories (*P* for trend < .001).

**Table 2:** Association between DI-GM and GSD, NHANES 2017–2020.

Exposures	Model 1*		Model 2**	
	OR (95% CI)	*P*-value	OR (95% CI)	*P*-value
DI-GM (continuous)	0.92 (0.87–0.97)	.002	0.92 (0.87–0.97)	.004
DI-GM categories				
0–3	Ref	–	Ref	–
4	0.83 (0.66–1.05)	.126	0.86 (0.68–1.1)	.245
5	0.76 (0.6–0.96)	.023	0.82 (0.64–1.04)	.11
≥6	0.68 (0.54–0.86)	.002	0.71 (0.55–0.91)	.007
*P* for trend		<.001		<.001
Beneficial to gut microbiota	0.92 (0.86–0.98)	.013	0.93 (0.86–0.99)	.047
Unbeneficial to gut microbiota	0.92 (0.85–1)	.07	0.9 (0.82–0.99)	.031
DII (continuous)	1.15 (1.07–1.23)	<.001	1.12 (1.04–1.21)	.003
DII tertiles				
T1	Ref	–	Ref	–
T2	1.24 (0.99–1.56)	.061	1.20 (0.95–1.53)	.120
T3	1.55 (1.22–1.96)	<.001	1.42 (1.10–1.83)	.006
*P* for trend		<.001		.002

CI = confidence interval, DI-GM = dietary index for gut microbiota, DII = Dietary Inflammatory Index, GSD = gallstone disease, NHANES = National Health and Nutrition Examination Survey, OR = odds ratio.

* Model 1 no adjusted.

** Model 2 adjusts for age, gender, PIR, ethnicity, diabetes, hypertension, body mass index, hypertension, diabetes, and smoking.

Examining the DI-GM components, the beneficial to gut microbiota score was inversely associated with GSD (crude: OR = 0.92, 95% CI: 0.86–0.98, *P* = .013; adjusted: OR = 0.93, 95% CI: 0.86–0.99, *P* = .047). The unbeneficial to gut microbiota score showed a significant association only in the adjusted model (OR = 0.90, 95% CI: 0.82–0.99, *P* = .031).

### 3.3. Sensitivity analyses

Table 3 presents the stratified analysis of the association between DI-GM and GSD based on age, gender, ethnicity, smoking status, and BMI. The findings demonstrate a consistent trend in the relationship between DI-GM and GSD risk across all subgroups. Moreover, no significant interactions were observed (*P* for interaction > .05), indicating that the effect of DI-GM on GSD risk remains relatively stable across different subgroups.

**Table 3:** Associations between DI-GM and GSD, stratified by selected factors, NHANES 2017–2020.

Characteristic	OR (95% CI)	*P*-value	*P* for interaction
Age (yrs)			.681
<40	0.94 (0.81–1.1)	.49	
40–59	0.91 (0.81–1.01)	.082	
≥60	0.9 (0.83–0.98)	.016	
Gender			.329
Male	0.9 (0.82–1)	.053	
Female	0.91 (0.85–0.98)	.02	
Ethnicity (%)			.54
Hispanic	0.98 (0.87–1.11)	.8	
Non-Hispanic White	0.87 (0.79–0.95)	.002	
Non-Hispanic Black	0.99 (0.86–1.13)	.88	
Other	0.9 (0.72–1.12)	.35	
Smoking status (%)			.427
Smoker	0.91 (0.81–1.02)	.13	
Nonsmoker	0.9 (0.84–0.97)	.005	
Body mass index (kg/m^2^)			.324
<24.9	0.96 (0.86–1)	.067	
25–29.9	0.97 (0.92–1.03)	.18	
≥30	0.93 (0.87–0.98)	.026	

Each stratification was adjusted for age + sex + ethnicity + marital status + poverty income ratio + hypertension + diabetes + BMI + smoking status+. The strata variable was not included when stratifying by itself.

BMI = body mass index, CI = confidence interval, DI-GM = dietary index for gut microbiota, GSD = gallstone disease, NHANES = National Health and Nutrition Examination Survey, OR = odds ratio, PIR = poverty income ratio.

**Figure 1.**
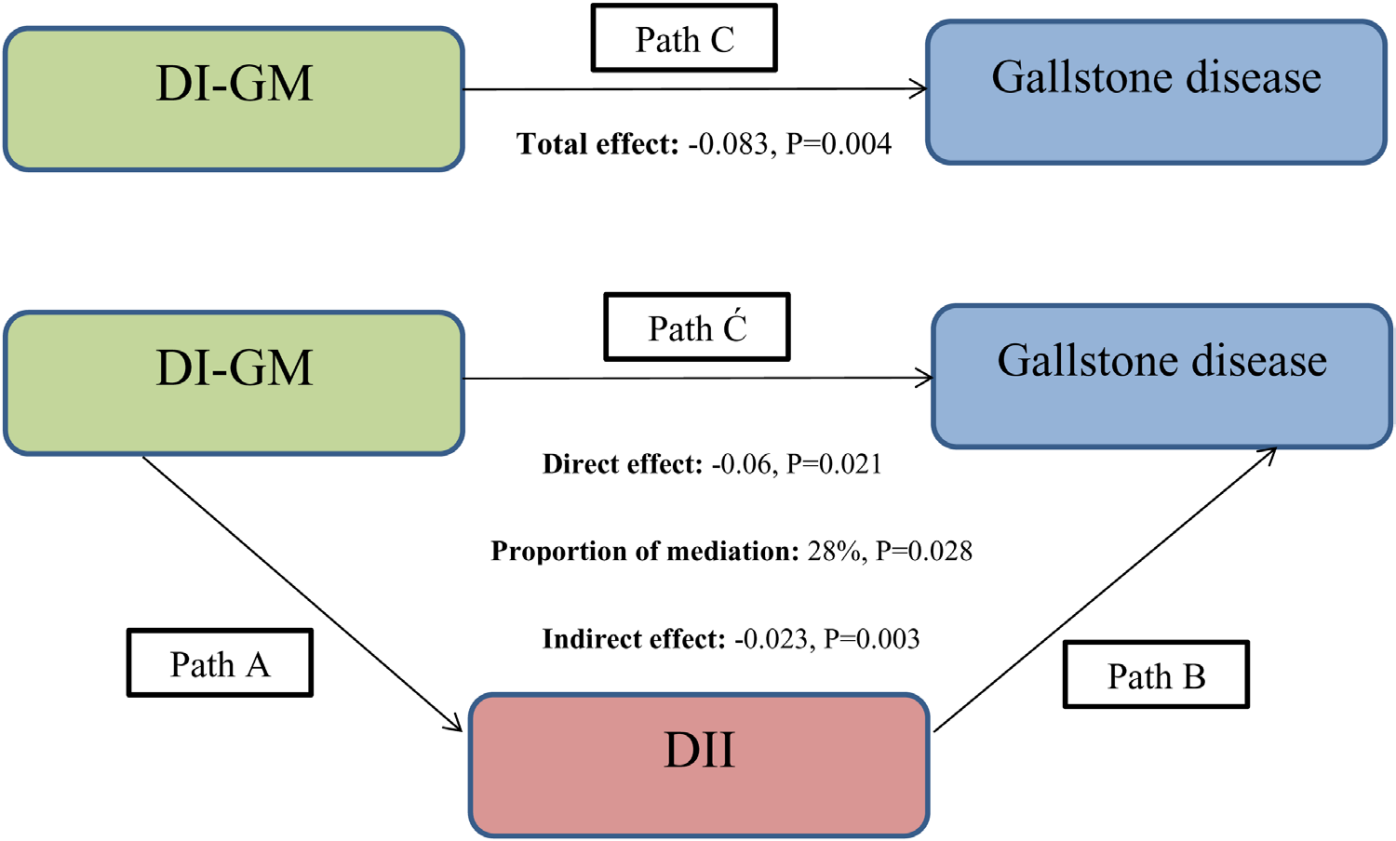
Schematic diagram of the mediation effect analysis. Path C indicates the total effect; path Ć indicates the direct effect. The indirect effect is estimated as the multiplication of paths A and B (path A × B). The mediated proportion is calculated as indirect effect/(indirect effect + direct effect) × 100%. Analyses were adjusted for age, gender, race, PIR, ethnicity, diabetes, hypertension, BMI, smoking, and physical activity. Mediation analysis was conducted to investigate whether the effect of DI-GM on GSD is mediated by DII. BMI = body mass index, DI-GM = dietary index for gut microbiota, DII = Dietary Inflammatory Index, GSD = gallstone disease, PIR = poverty income ratio.

The mediation model and pathways are illustrated in Figure 1, with DI-GM as the independent variable, GSD as the dependent variable, and DII as the mediating variable. As shown in Table 4, a significant association was observed between DI-GM and DII after adjusting for covariates (β = −0.45, 95% CI: −0.49 to −0.41, *P* < .001). After adjusting for all covariates, the mediating effect of DII was identified (Fig. 1), with an indirect effect of − 0.023 (*P* = .003) and a direct effect of − 0.06 (*P* = .021). The mediated proportion was 28% (*P* = .028). Therefore, DII can be considered a partial mediator in the association between DI-GM and the likelihood of GSD, indicating that a gut microbiota-favorable diet may reduce GSD risk in part through lowering dietary inflammation.

**Table 4:** Multivariate linear regression of DI-GM and DII.

	β	95% CI	*P*-value
DI-GM–DII	−0.45	−0.49 to −0.41	<.001

Adjusted for age, gender, race, PIR, ethnicity, diabetes, hypertension, BMI, smoking, and physical activity.

BMI = body mass index, CI = confidence interval, DI-GM = dietary index for gut microbiota, DII = Dietary Inflammatory Index.

The mediation analysis suggests that DII may partially explain the association between DI-GM and GSD; however, this finding should be interpreted as an exploratory indirect association rather than evidence of causal mediation, given the cross-sectional design and lack of temporal sequencing.

Moreover, the results from additional adjustment, multiple imputation, and unweighted association analyses confirmed that the association between DI-GM and GSD remained significant and robust (Table S2, Supplemental Digital Content 2).

## 4. Discussion

This study analyzed data from 5669 participants in the 2017–2020 NHANES to investigate the association between the DI-GM and GSD risk. The findings revealed that higher DI-GM scores, indicative of diets more beneficial to gut microbiota, were inversely associated with GSD presence. Specifically, individuals with DI-GM scores of 5 and 6 had lower odds of GSD compared to those with scores of 0 to 3. Stratified analyses showed no significant interaction among different subgroups. Overall, these results suggest a potential beneficial association between microbiota-related dietary patterns and GSD prevalence. Our mediation findings showed that DII partially mediates the association between the DI‑GM and GSD.

Baseline analysis showed that patients with GSD were older, which aligns with existing literature indicating that the prevalence of gallstones increases with age.^[14]^ Aging has been associated with physiological changes such as reduced gallbladder motility and altered lipid metabolism.^[3,15]^ Gender distribution showed a higher prevalence of GSD among females than males. Female sex hormones, particularly estrogen and progesterone, are often implicated as key factors in explaining gender differences in the prevalence of GSD.^[3]^ Ethnic disparities were evident, with Non-Hispanic Whites representing a higher proportion than in other ethnicities. Genetic predisposition as well as Western dietary patterns can be associated with increased GSD risk in this population.^[16]^ Additionally, participants with GSD exhibited higher rates of hypertension and diabetes. GSD, hypertension, and diabetes are components of metabolic syndrome, which is characterized by insulin resistance, central obesity, dyslipidemia, and elevated blood pressure. Insulin resistance can lead to increased hepatic secretion of cholesterol, resulting in supersaturation of bile and gallstone formation.^[17]^ It is hypothesized that hypertension may alter bile composition or affect gallbladder motility, contributing to gallstone formation.^[18]^ Based on common associations between obesity and GSD, the mean BMI was lower in the non-GSD group compared to the GSD group.^[19]^

The inverse association between DI-GM and GSD is consistent with prior studies reporting relationships between dietary quality and gallstone prevalence. For instance, a study utilizing NHANES data found that higher levels of the oxidative balance score, reflecting a diet rich in antioxidants, were associated with lower odds of GSD.^[20]^ Similarly, research on lipid accumulation products and GSD risk also underscores the association between abdominal obesity and GSD.^[21]^ In another study, a higher DII and Healthy Eating Index-2020, lower Alternative Healthy Eating Index, Mediterranean diet, and dietary approaches to stop hypertension, were all positively associated with increased GSD prevalence.^[7]^ Other similar studies confirmed the significant association between DII, Composite Dietary Antioxidant Index and GSD.^[8,9]^ As our knowledge, there is no study investigating the association between the DI-GM and GSD risk in US adults.

Our mediation findings, showing that DII partially mediates the association between the DI‑GM and GSD, are consistent with and extend prior research linking diet, inflammation and gallstones. For example, a cross‑sectional analysis of NHANES data reported that higher DII was significantly associated with increased odds of GSD.^[22]^ Meanwhile, systematic reviews have shown that higher DII scores correlate with unfavorable gut microbiota profiles (lower diversity, reduced short-chain fatty acids producers) which in turn promote intestinal and systemic inflammation.^[23]^ Gong et al found that DII had a mediatory role in the association between DI-GM and sarcopenia.^[24]^ However there is no study investigating this relationship in GSD.

The inverse association between DI-GM and GSD observed in this study is consistent with prior epidemiological evidence linking healthier dietary patterns to lower GSD prevalence.^[7]^ Previous studies using NHANES data and other population-based cohorts have reported that dietary indices reflecting anti-inflammatory or antioxidant properties are associated with reduced gallstone risk.^[9]^ Our findings extend this literature by suggesting that dietary patterns supportive of gut microbiota may also be associated with GSD prevalence. Importantly, the associations observed in this study are based on epidemiological analysis and should be interpreted independently from hypothesized biological mechanisms, which require confirmation through experimental and longitudinal studies.

Utilizing NHANES data provides a diverse and nationally representative sample, enhancing the generalizability of the findings. The use of 24-hour dietary recall data allows for a detailed evaluation of dietary patterns and their association with gallstone prevalence. The study accounted for multiple potential confounders, including age, gender, race, socioeconomic status, and comorbidities, strengthening the validity of the results. However, some limitations must be noted. First, the cross-sectional design precludes the establishment of temporal relationships between DI-GM scores and GSD, and therefore causal inference cannot be made. Second, GSD status was based on self-reported physician diagnosis, which may be subject to misclassification bias due to recall error or undiagnosed cases. Third, dietary intake was assessed using two 24-hour dietary recalls, which may not fully capture habitual dietary intake and is subject to recall bias and within-person variation. Fourth, despite adjusting for multiple covariates, residual confounding may remain due to unmeasured factors such as medication use, genetic susceptibility, physical activity, and detailed clinical history. Fifth, findings on DII mediatory role should be interpreted as exploratory indirect associations rather than causal mediation effects, as temporal relationships between DI-GM, DII, and GSD cannot be established. Longitudinal studies are required to properly evaluate mediation effects over time. Finally, the absence of direct gut microbiota measurements limits the ability to confirm specific microbial profiles underlying the observed associations between DI-GM and GSD.

## 5. Conclusion

Individuals with DI-GM scores of 5 and 6 had lower odds of GSD compared to those with scores of 0 to 3. Stratified analyses showed no significant interaction among different subgroups. These findings indicate an inverse association between dietary patterns favorable to gut microbiota and GSD prevalence in a U.S. adult population. However, given the cross-sectional design of this study, causal relationships cannot be inferred. Further prospective cohort studies and longitudinal investigations are needed to confirm these associations and clarify the temporal and potential biological relationships between dietary patterns and GSD. Mediation analysis showed that DI-GM was inversely associated with DII, and a statistically significant indirect association through DII was observed. However, given the cross-sectional design, these findings should be interpreted as exploratory indirect associations rather than causal mediation effects, as temporal relationships between DI-GM, DII, and GSD cannot be established.

## Acknowledgments

We would like to thank all NHANES staff and participants.

## Author contributions

**Conceptualization:** Changqing Deng, Yijin Chen, Wenwen Ni.

**Data curation:** Changqing Deng, Wenwen Ni.

**Formal analysis:** Yijin Chen, Wenwen Ni.

**Methodology:** Wenwen Ni.

**Writing – original draft:** Changqing Deng, Yijin Chen, Wenwen Ni.

**Writing – review & editing:** Changqing Deng, Yijin Chen, Wenwen Ni.




