# Supplementary material for: Association between the dietary index for gut microbiota and gallstone disease: A cross-sectional analysis considering the Dietary Inflammatory Index
Source: Medicine (Baltimore). 2026 Jul 31;105(31):e49813. doi: 10.1097/MD.0000000000049813 (PMC13433036; doi:10.1097/MD.0000000000049813)
Supplement: Supplementary file 1 [file medi-105-e49813-s001.docx]

| **Supplemental table S1. DI-GM Components and Scoring Criteria** | | | |
| --- | --- | --- | --- |
| **Component** | **Unit** | **Sex-Specific Median Cut-Point*** | **Scoring Criteria** |
| **Beneficial Components** | | | |
| Whole grains | oz equivalents/day | 0.65 | 1 point if > median |
| Avocados | servings/day | 0.10 | 1 point if > median |
| Cranberries | servings/day | 0.05 | 1 point if > median |
| Chickpeas | servings/day | 0.20 | 1 point if > median |
| Broccoli | servings/day | 0.15 | 1 point if > median |
| Soybean | servings/day | 0.08 | 1 point if > median |
| Fermented dairy | servings/day | 0.25 | 1 point if > median |
| Fiber | g/day | 14.2 | 1 point if > median |
| Coffee | servings/day | 1.50 | 1 point if > median |
| Green tea | servings/day | 1.50 | 1 point if > median |
| **Unbeneficial Components** | | | |
| Refined grains | oz equivalents/day | 4.80 | 1 point if < median |
| Red meat | servings/day | 1.20 | 1 point if < median |
| Processed meat | servings/day | 1.20 | 1 point if < median |
| Very high-fat diet** | % | 35.0 | 1 point if < median |
| *Sex-specific median cut-points derived from the full NHANES 2017-2020 **Defined as >35% of total energy from fat based on dietary guidelines | | | |
