# Supplementary material for: Association between the dietary index for gut microbiota and gallstone disease: A cross-sectional analysis considering the Dietary Inflammatory Index
Source: Medicine (Baltimore). 2026 Jul 31;105(31):e49813. doi: 10.1097/MD.0000000000049813 (PMC13433036; doi:10.1097/MD.0000000000049813)
Supplement: Supplementary file 2 [file medi-105-e49813-s002.docx]

Supplementary table

| **Supplemental table S2. Sensitivity analyses for the association between DI-GM and GSD** | | | | |
| --- | --- | --- | --- | --- |
| **Exposure** | **Main Analysis OR (95% CI)** | **Multiple Imputation OR (95% CI)** | **Unweighted Analysis OR (95% CI)** | **Additional Adjustment* OR (95% CI)** |
| DI-GM (continuous) | 0.92 (0.87–0.97) | 0.91 (0.86–0.97) | 0.93 (0.88–0.98) | 0.91 (0.86–0.97) |
| DI-GM ≥6 vs 0–3 | 0.71 (0.55–0.91) | 0.69 (0.53–0.89) | 0.74 (0.58–0.94) | 0.69 (0.53–0.89) |
| Beneficial component | 0.93 (0.86–0.99) | 0.92 (0.86–0.99) | 0.94 (0.88–1.00) | 0.92 (0.86–0.99) |
| Main analysis adjusted for age, gender, race/ethnicity, poverty income ratio, body mass index, smoking status, diabetes, and hypertension. *Additional adjustment model further included total energy intake, physical activity, marital status and alcohol consumption. Multiple imputation was performed using chained equations with five imputed datasets, and pooled estimates were calculated using Rubin’s rules. Unweighted analyses were conducted without applying NHANES sampling weights, strata, or clusters. Abbreviations: GSD, gallstone disease; DI-GM, Dietary Index for Gut Microbiota; NHANES, National Health and Nutrition Examination Survey; OR, odds ratio; CI, confidence interval. | | | | |
